# A Semantic-Enabled Platform for Realizing an Interoperable Web of Things

**DOI:** 10.3390/s19040869

**Published:** 2019-02-19

**Authors:** Jorge Lanza, Luis Sánchez, David Gómez, Juan Ramón Santana, Pablo Sotres

**Affiliations:** 1 Network Planning and Mobile Communications Lab, University of Cantabria, 39012 Santander, Spain; jlanza@tlmat.unican.es (J.L.); jrsantana@tlmat.unican.es (J.R.S.); psotres@tlmat.unican.es (P.S.); 2 Atos Research & Innovation, C/ Albarracín 25, 28037 Madrid, Spain; david.gomez@atos.net

**Keywords:** interoperability, Web-of-Things, semantics, Internet-of-Things, registry

## Abstract

Nowadays, the Internet of Things (IoT) ecosystem is experiencing a lack of interoperability across the multiple competing platforms that are available. Consequently, service providers can only access vertical data silos that imply high costs and jeopardize their solutions market potential. It is necessary to transform the current situation with competing non-interoperable IoT platforms into a common ecosystem enabling the emergence of cross-platform, cross-standard, and cross-domain IoT services and applications. This paper presents a platform that has been implemented for realizing this vision. It leverages semantic web technologies to address the two key challenges in expanding the IoT beyond product silos into web-scale open ecosystems: data interoperability and resources identification and discovery. The paper provides extensive description of the proposed solution and its implementation details. Regarding the implementation details, it is important to highlight that the platform described in this paper is currently supporting the federation of eleven IoT deployments (from heterogeneous application domains) with over 10,000 IoT devices overall which produce hundreds of thousands of observations per day.

## 1. Introduction

The Internet of Things (IoT) is unanimously identified as one of the main technology enablers for the development of future intelligent environments [1]. It is driving the digital transformation of many different domains (e.g., mobility, environment, industry, healthcare, etc.) of our everyday life. The IoT concept has attracted a lot of attention from the research and innovation community for a number of years already [2,3,4]. One of the key drivers for this hype towards the IoT is its applicability to a plethora of different application domains [5], like smart cities [6,7], e-health [8,9], smart-environment [10,11], smart-home [12], or Industry 4.0 [13]. This is happening by realizing the paradigm of more instrumented, interconnected, and intelligent scenarios, which are instrumented through low-cost smart sensors and mobile devices that turn the workings of the physical world into massive amounts of data points that can be measured. Interconnected so that different parts of a core system, like networks, applications, and data centers, are joined and “speak” to each other, turning data into information. Finally intelligent, with information being transformed into real-time actionable insights at massive scale through the application of advanced analytics.

“Today, there are roughly 1.5 billion Internet-enabled PCs and over 1 billion Internet-enabled smartphones. The present ‘Internet of PCs’ will move towards an ‘Internet of Things’ in which 50 to 100 billion devices will be connected to the Internet by 2020. Some estimations point to the fact that in the same year, the amount of machine sessions will be 30 times higher than the number of mobile person sessions” [14]. The IoT has drastically changed some of the key challenges that Future Internet will have to address. Until recently, researchers have focused on devices and the communication technologies used to access them. However, as it happened with nowadays Internet, most of the revenue is projected to come from the services that can be provided using all these devices and communication networks as a basis. While billions of devices are connecting to the Internet, as it happened with “Internet of PCs”, the Web has emerged again as the paradigm to exploit the potential of the myriad of connected devices in so-called cyber physical environments. The Web of Things (WoT) paradigm is emerging with virtual representations of physical or abstract realities increasingly accessible via web technologies. Achieving a new phase of exponential growth, comparable to the earliest days of the Web, requires open markets, open standards, and the vision to imagine the potential for this expanding WoT.

This paper describes the design and implementation of a solution to enable the WoT paradigm. The so-called Semantic IoT Registry (IoT Registry at GitHub (https://github.com/tlmat-unican/fiesta-iot-iot-registry)) consists on a semantic web-enabled repository that provides platform independent APIs for application developers as well as the baseline for different platforms to interoperate with one another. The approach taken is based upon semantically enriched metadata that describes the data and interaction models exposed to applications. In this sense, it enables platforms to share the same meaning when they share their data through the IoT Registry. This data is available as Linked Data on Resource Description Framework (RDF) format through RESTful APIs. In addition to the support of semantically enriched data, which is the basis for fulfilling the data interoperability requirement, the IoT Registry enables the use of Uniform Resource Identifiers (URIs) as addresses for things serving as proxies for physical and abstract entities. This latter aspect is of utmost importance for realizing the infrastructure-agnostic scenario that a WoT comprising cross-domain platforms requires. 

The IoT Registry is the base component underpinning the application development across federated IoT data sources through the provision of Application Programming Interfaces (APIs) to get and push data from any of the underlying IoT infrastructures. Thus, it is important to highlight that the implementation details presented in this paper are the result of overcoming the challenges, mainly in terms of heterogeneity and scalability, that the federation of eleven IoT deployments (from heterogeneous application domains), with over 10,000 IoT devices overall which produce hundreds of thousands of observations per day [15], poses on the implemented system.

The remainder of the article is structured as follows. Section 2 makes a non-exhaustive review of relevant related work in the key research topics that underpin the IoT Registry. Section 3 introduces the key design considerations that have been taken into account for the development of the IoT Registry. The detailed description of the IoT Registry architecture and its functional building blocks is presented in Section 4. Section 5 goes a level deeper and focus on the implementation details for the storage and distribution of semantic data streams paying special attention to the solutions used to address the challenges of scalability and resource abstraction. Finally, Section 6 concludes the document and briefly discusses those open issues that shall be addressed in the future.

## 2. Related Work

### 2.1. Semantic IoT Interoperability

Currently fragmented IoT ecosystem is jeopardizing the development of global solutions. The existing multiple parallel IoT platforms have to converge towards offering seamless, global, and linked services to their users. It is necessary to implement solutions that are able to make the already existing IoT infrastructures to collaborate in providing a common and portable way of offering their data services. One of the aims of the platform described in this article is to support the automation of the deployment of services/applications over heterogeneous IoT domains.

Semantic technologies will play a key role to align the descriptions of the various IoT entities from various platforms. However, defining the abstraction level required for an IoT ontology is challenging. Nowadays, there are plenty of initiatives that are specifying the models to represent IoT devices and the ecosystem around them. Nonetheless, it is still difficult to find one that addresses all the requirements. Probably, the most widely used ontology is the Semantic Sensor Network (SSN) Ontology [16] that covers sensing, but does not take actuating or other realms of IoT into account. Moreover, this ontology is very complex to use at its full extension and is typically used as a baseline reference. The IoT-Lite ontology [17] uses the SSN as a basis and adds Architectural Reference Model (ARM) [18] key concepts to provide a more holistic IoT model. The adopted solution within FIESTA-IoT has been to reuse these generic ontologies and extend them wherever required to meet the requirements identified for the federation of IoT testbeds.

Other works that are pursuing parallel objectives and are worth mentioning are OneM2M [19] or IoT-O [20]. OneM2M, as an international partnership project of standardization bodies, is defining a standard for M2M/IoT-communications. The actual release is lacking semantic description of resources that will be addressed as one major point in the next release. The IoT-O ontology aims to unify different ontologies in the IoT landscape and consists of eight different ontologies. It is actively maintained and connected to other standardizations like OneM2M. While many similarities can be found with the FIESTA-IoT ontology, geolocation of resources and observations is not addressed and the virtual entity concept that is central for the IoT-A ARM is not properly covered. Moreover, to the best of our knowledge no major IoT platforms have already adopted it for semantically handling its resources and observations. Other ontologies are focusing on either specific subdomains, like the Sensor Web for Autonomous Mission Operations (SWAMO) ontology [21], which concentrates on marine interoperability or not specifically defined for the IoT domain like GoodRelations [22] that is dealing with products but can be taken into account in the industrial IoT area. The Smart Appliance REFerence (SAREF) ontology [23] is another initiative which is raising much attention as enables semantic interoperability for smart appliances. It has been created in close interaction with the industry, thus, fostering the future take up of the modeling defined. Still, it is centered on home appliances that might make it fall short in other domains.

### 2.2. RDF Description and Validation

Semantics define a powerful take on how to shape, allot, catalogue, and share information. Moreover, it is possible to construct a distributed database accessible from any Internet-connected device by using URIs as identifiers and locators for the different pieces of information. However, it is necessary to guarantee that all the information stored in this database complies with the rules established in the ontology that underpins the semantic nature of that data. Otherwise, it would not be of actual value, as it would be weighed down by issues related to data quality and consistency.

Recent approaches to validate RDF—the most common format for representing semantic data—have been proposed. ShEx [24] and SHACL [25] are languages for describing RDF graph structures so that it is possible to identify predicates and their associated cardinalities and data types. Both of them have the same objective, to enable validating RDF data by using a high-level language. Both define the notion of a shape, as the artifact to specify constraints on the topology of RDF nodes. SHACL shapes are analogous to ShEx shape expressions, with the difference that links to data nodes are expressed in SHACL by target declarations and in ShEx by shape maps. In most of the common cases, it is possible to translate between ShEx and SHACL. The triples involving nodes in the graph that must be validated have to comply with the defined shapes. If not matching, the RDF graph would not be consistent with the semantic model that wants to be used.

The IoT registry imposes RDF validation before storing the datasets that arrives from the underlying IoT platforms. The solution adopted in the reference implementation of the IoT registry did not make use of any of the aforementioned languages due to some practical reasons. Firstly, SHACL recommendation only appeared on July 2017 [25], but the implementation of the IoT registry started much before. The first works started on 2015, but they were only working drafts. Moreover, available implementations, both for SHACL and ShEx, are under development. Secondly, the FIESTA-IoT ontology, which was the one that we used for our reference implementation, did not have the shapes graph that is necessary to apply SHACL or ShEx validators. Finally, yet critically important, we preferred to address the validation process by directly applying SPARQL filtering in order to avoid further delays, associated with the execution of shapes validation procedure and the evaluation of its validation report, in the processing of incoming data.

### 2.3. Web of Things

The WoT emerges from applying web technologies to the IoT to access information and services of physical objects. In WoT, each physical object possesses a digital counterpart. These objects are built according to Representational state transfer (REST) architecture and accessed with HTTP protocol via RESTful API. A Web Thing can have an HTML or JSON representation, REST API to access its properties and actions, and an OWL-based semantic description.

W3C has recently launched the Web of Things Working Group [26] to develop initial standards for the Web of Things. Its main aim is “to counter the fragmentation of the IoT”. They are still working on defining the WoT architecture and the description of the WoT Thing, which should define a model and representation for describing the metadata and interfaces of Things, where a Thing is the virtualization of a physical entity that provides interactions to and participates in the WoT.

In parallel to this standardization effort, several projects and platforms have been developed targeting the support of service provision based on the WoT paradigm. Paganelli et al. [27] present their WoT-based platform for the development and provision of smart city services. Precision agriculture is the application domain that benefits from the platform described by Foughali et al. [28]. While they provide some of the solutions promised by the WoT, still do not address the IoT fragmentation as they rely on proprietary modeling. Other works [29,30,31], instead, leverage semantic technologies to fulfill the extendable modeling requirement. As we are proposing in this paper, we believe that this is the necessary combination in order to fully develop the WoT concept into a running system. The key novelty from the work presented in this paper is that previous works have not been implemented and proven over real-world scenarios with federation of heterogeneous IoT infrastructures, as it is the case of the platform presented in this paper.

### 2.4. RDF Streams

Annotating data following RDF principles is the first step for enabling interoperability. However, it still necessary to access to this data so that services can be provided on the basis of the context knowledge that it enables. IoT data is typically associated to the Big Data paradigm. However, it is not only large in volume. An important difference between the IoT datasets compared to the conventional ones is the quick changes in data and dynamicity of the environment.

Different RDF Stream Processing (RSP) systems have been proposed to enable querying over RDF streams, in opposition to the typical static querying. Most of them are extensions of SPARQL that take into account the dynamicity of the data that is being queried [32]. 

The solution implemented in the IoT Registry has some similarities with RSP but the key difference is that IoT registry design considerations make it to be settled somehow in between the persistent and transient data. Most of the time, only the latest knowledge about a dynamic system that is being monitored is important. However, access to history is also necessary for some applications. IoT Registry tries to provide a solution for both demands. This is, facilitate the access to the most recent data but also enable access to relevant information in the past.

### 2.5. IoT Platforms Federation

The vision of integrating IoT platforms and associated silo applications is bound to several scientific challenges, such as the need to aggregate and ensure the interoperability of data streams stemming from them. The convergence of IoT with cloud computing is a key enabler for this integration and interoperability. It facilitates the aggregation of multiple IoT data streams so that it is possible to develop and deploy scalable, elastic, and reliable applications that are delivered on demand according to a pay-as-you-go model. During the last 4–5 years several efforts towards IoT/Cloud integration have been proposed [33,34] and a wide range of commercial systems (e.g., Xively [35] and ThingsSpeak [36]) are available. These cloud infrastructures provide the means for aggregating data streams and services from multiple IoT platforms. Moreover, other initiatives, such as Next Generation Services Interface (NGSI) [37] promoted by Open Mobile Alliance (OMA) or Hyper/Cat [38], focus on the homogenization of interfaces enabling web access to data produced by IoT deployments. However, they are not sufficient for alleviating the fragmentation of IoT platforms. This is because they emphasize on the syntactic interoperability (i.e., homogenizing data sources, interfaces, and formats) rather than on the semantic interoperability of diverse IoT platforms, services, and data streams. They intentionally enforce no rules about metadata naming. Use of a reference ontology is one of the currently explored possibilities for having interoperable systems.

Some open source initiatives like Project Haystack [39], which aims at streamlining the access to data from the IoT, or the IoT Toolkit [40], which has developed a set of tools for building multiprotocol Internet of Things gateways and service gateways, are working on standardization of semantic data models and web services. Their goal is making it easier to unlock value from the vast quantity of data being generated by the smart devices that permeate our homes, buildings, factories, and cities. In this sense, the differential aspect of the platform proposed in this paper is that it is taking a holistic approach, considering the semantic federation of testbeds as a whole, vertically and horizontally integrated, and independently of the environment (smart city, health, vehicles, etc.) they are controlling.

## 3. Semantic IoT Registry Design Considerations

In this section, we are reviewing the key requirements that have defined the main design considerations that we have observed during the development of the Semantic IoT Registry. This review is important in order to understand the design choices and the solution’s architecture and building blocks, which are described in the next sections.

Interoperability: Frequently denoted as interworking, it characterizes the capacity of systems to connect and interchange data. In the IoT domain, where many vendors will coexist, access to information from diverse platforms is essential in order to not be trapped in vendor lock-in situations. Interoperability entails for conversion between systems and sharing data via well-known interfaces in order to facilitate its exploitation. Two, among the different options that can be considered, are particularly interesting:

- To replicate IoT data, leveraging the existing knowledge on the existing platforms. With this option, the response time would not be affected if new nodes were added. However, this solution requires higher capacity, which could lead to scalability problems if the data set grows in an unbounded way.
- To discover and translate the IoT data in real time. In this option, the home IoT platforms are used to retrieve the information, thus avoiding duplication, hence adapting not only the underlying IoT data but also the operations allowed by the different interfaces. On the cons side, this would introduce extra complexity and the increase in the overall system response time.

IoT data abstraction and integration: Data abstraction in IoT is related to the way in which the physical world is represented and managed. It is necessary to have a set of concepts to properly describe not only the IoT device itself but also the sensor observation and measurement data. Using semantic descriptions, the IoT data can be homogeneously characterized. However, it is necessary to employ a generic model that is capable of supporting the mapping among the used semantic models that might exist in a cross-platform IoT integration scenario. Thus, the solution has to focus on a canonical set of concepts that all IoT platforms can easily adopt. We opted for taking as reference the IoT Architecture Reference Model (ARM) as defined in the IoT-A project [18]. The foremost aspect that this choice implies is that the ontology that is used to regulate the semantic annotation of the testbeds’ resources is only bound by the core concepts that compose the aforementioned ARM Domain and Information Models. These core concepts are

- A Resource is a “Computational element that gives access to information about or actuation capabilities on a Physical Entity” [18].
- An IoT service is a “Software component enabling interaction with IoT resources through a well-defined interface” [18].

These concepts conform the baseline for representing the devices and overall IoT infrastructure. However, there is still a major concept that is not tackled within the ARM models. This concept relates to the actual data that is gathered by the devices and offered through the services that expose them. It is called the Observation concept:

- An Observation is a “piece of information obtained after a sensing method has been used to estimate or calculate a value of a physical property related to a Physical Entity.”

Data semantization: Besides the actual technologies used for exporting the data services, the main feature that underpins our solution is the fact that the information is exchanged in a semantically annotated format. The IoT Registry is not bound to any ontology in particular so its design is fundamentally reusable and extendable. This means that while using a common ontology is necessary to make it possible to seamlessly deal with data from different sources, the design of the IoT Registry does not specify the ontology that has to be used. Additionally, from the data representation viewpoint, a specific RDF representation formats (e.g., RDF/XML, JSON-LD, Turtle, etc.) should not be imposed. Data consumers should be able to select their preferred format in every request. Thus, all of them must be supported.

Cyberphysical Systems as a Service (CPSaaS): The value of IoT is on the ability to ease the access to the services that the IoT devices embedded in our surroundings export. IoT is typically reduced to the data that sensors can produce but it is not only that. IoT is an integral part of Cyberphysical Systems (CPS) and vice versa. One of the key design considerations of the IoT Registry is that not only data but also services have to be interoperable. The CPSaaS consideration relates precisely with the ability to access the services exported by IoT and/or CPS platforms in an interoperable and platform agnostic manner. Thus, it is necessary to guarantee that application developers, which are the ones that at the end will consume those services through their applications, can use these services in the most user-friendly manner. Nowadays, REST APIs are the de facto standard for exporting services. Thus, it is required that access to datasets, data streams, and IoT Services in general is orchestrated through REST-based interfaces. Additionally, the heterogeneity of the underlying infrastructure should be hidden to the final data and service consumers. This way, the interoperability is something that the platform has to guarantee but, at the same time, has to be simply offered to the end user. That is, they will consume data and services based on their interest and necessities but without even knowing if it comes from one or many different platforms.

Services Exportability: The challenges that IoT-enabled smart solutions face are very similar among them. Hence, the context-aware, intelligent services that are meant to optimize the scenarios to which they are applied are potentially beneficial to any of them regardless of its location (e.g., services for improving the efficiency of the city and the well-being of its citizens are applicable to any city). This is one of the key motivating factors for service providers and application developers to invest resources in designing and implementing such services. Nonetheless, if deploying the same service in different cities is not straightforward, the profit margins are put at risk due to the cost of tailoring the service to the infrastructure and platform that is deployed in each city. Only if the same service or application can be seamlessly used across cities, the full potential of IoT-based services will be reached.

Access to history: IoT is typically assumed to have just a stream nature. That is, only the current situation is important. As new observations from one sensor arrive, the previous measurements can be discarded. This is true in most of the cases but the value of accessing historical information cannot be neglected. Combination of IoT datasets and BigData algorithms are used for critical applications that are based on the identification of patterns, for which storage of historical records is fundamental.

Discoverability: It is not enough with setting the mechanisms to make data interoperable among IoT Platforms, but it is also necessary to fetch this data from the repositories were it is stored. Due to the huge variety of information and domains that can coexist, it is necessary to have an elastic discovery environment that can exploit the richness of the semantic-enabled information stored.

Programmability: Creating the ecosystem is the necessary but not sufficient condition. A fundamental risk of such ecosystem is that developers and platform providers might find the ecosystem’s features unattractive. For the ecosystem to grow, developers must find it easy to interact with the interfaces offered. Enabling REST-based interfaces guarantee that in both cases interactions are straightforward.

## 4. Semantic IoT Registry Architecture

The IoT Registry is responsible of storing and managing the semantic descriptions that underlying IoT infrastructures make available. Considering that aggregated stored data can be of a different nature, the component must handle their differences while processing it homogeneously, to satisfy the needs of external users requesting information. In that sense, we mainly consider two tightly bound realms: the one related to the description of the resources belonging to the testbeds and the one related to the observations or measurements constantly gathered by those devices. Both of them are filled with semantic documents using the well-known RDF [41] serialization format. Thus, the core of the IoT registry component is composed of a Triplestore Database (TDB) and a fully-fledged API that allows the interplay with users.

**Figure 1:**
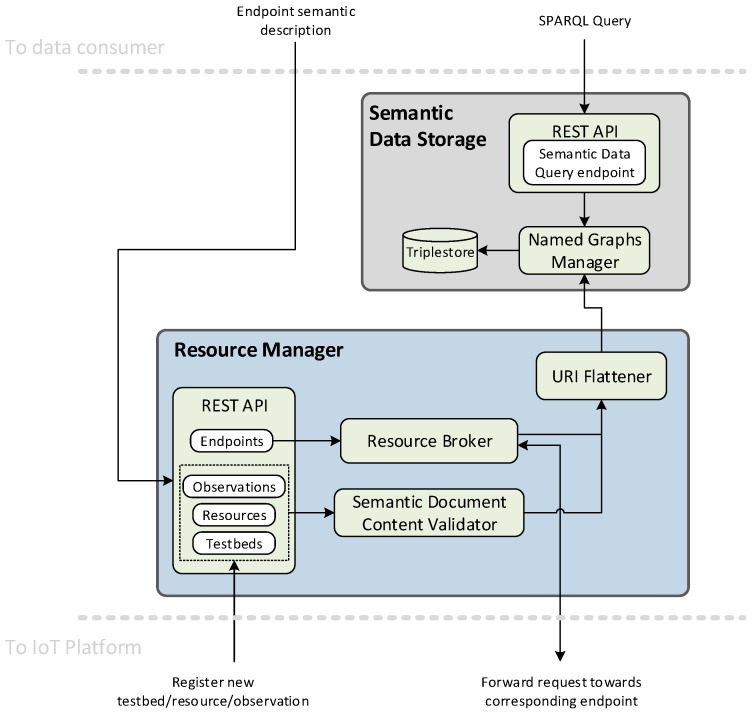
Internet of Things (IoT) registry functional architecture.

In addition to the TDB, the IoT registry is composed of a data query endpoint supported by a SPARQL engine, a manager that supervises IoT data registration and exposes the stored information to authorized users using a REST API, and an access broker that securely proxies direct access to registered resources. Figure 1 shows the internal architecture and the relationships between the different building blocks.

Although IoT Registry can run integrated within a more complex architecture [42], it can operate in standalone mode, providing basic authenticated access.

In the following sections, we describe in detail the main insights achieved from the implementation of these functional components, paying special attention to those features that make them suitable for addressing the challenges and design consideration brought about previously.

### 4.1. Semantic Data Storage

Because of the canonical concepts that underpin the model for data abstraction and semantization, the data stored at the IoT registry can be catalogued in two different, but closely interrelated, domains: Firstly, the descriptions of the IoT devices that are deployed in the field and, on the other hand, the measurements that they generate. The internal structure of the TDB follows a similar approach.

Before delving deeper into the TDB description, it is important to introduce the concept of Graph, which is basic for semantics and key to understand the remainder of the paper. A semantic graph is a network that represents semantic relationships between concepts, where the nodes of the network are the vertices of the graph, which represent the concepts, and the links of the network are the edges of the graph, which represent semantic relations between concepts. TDBs are said to store graphs since they store RDF triplets consisting of two nodes (concepts) and one link (the relation among them). When triplets containing nodes that were present in previously saved triplets are stored at the TDB they extend the graph whether creating new relations (i.e., the two nodes in the triplet were already in the graph) or introducing new concepts (i.e., one of the two nodes was not in the graph before). Additionally, inner graphs can be configured in TDBs in order to optimize its performance. These graphs focus on specific preconfigured concepts. This way, the information can be structured on different realms so that operations (e.g., store and fetch) that only affect one of these realms can be handled within the inner graph.

**Figure 2:**
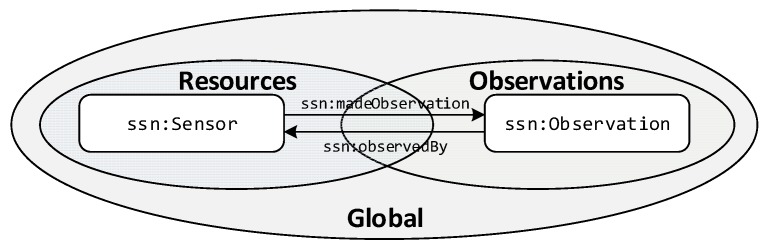
IoT registry TDB internal structure.

Based on the two identified realms, testbeds and its resources, and the observations collected in Figure 2, we introduced the high-level organization of the TDB. The approach followed for the IoT registry TDB considers two graphs, one for resources and another for observations’ descriptions. This provides a basic structure to the information stored, making it possible to focus the target when requesting or storing data. The dataset is also configured so that the default graph (named as Global in Figure 2) is defined to be the union of the two. The identifier of the sensor that made the observation is the link between the two realms. From the semantic point of view, the subjects and objects related via the properties *ssn:madeObservation* and/or *ssn:observedBy* are *ssn:Observation* and *ssn:Sensor*. As the sensor is referenced in both realms, the inference engine can establish the required relationships. As it has been previously mentioned, the IoT Registry design is not bound to any ontology; however, for the sake of clarity in the description we are using the concepts and relations from the FIESTA-IoT ontology [43], which is the one that we employed for the reference implementation that we have developed.

Although this was a design decision, the experience from real usage of our reference implementation has proven it was appropriate. Even when the users could always use the Global graph so that both graphs are used seamlessly on-the-fly, it was quite straightforward for them to direct their queries to only one domain. For example, they request either for a particular device’s property (i.e., location, physical phenomenon, or just metadata) inquiring only the Resources graph, or for the value and unit of measurement of an observation requiring information just from the Observations graph. The solution adopted offers flexibility and optimization. Performance-wise, shrinking the size of the targeted information reduces the expected response time. Consequently, queries should be adapted and run on either graphs based on the required information, resulting on a better user experience.

Last but not least, it is important to highlight that the TDB can be deployed in a distributed manner. This way, when queries are sent to the central IoT Registry, they will not only handle the request with its local TDB, but also forward it to the remote semantic query endpoint. This distributed mechanism avoids data replication and enables potential query parallelization, leading to a better response time. It is important to note that all the semantic databases should have the information modeled following the same ontology.

### 4.2. Resource Manager

The Resource Manager (RM) main objective is to supervise how IoT data is registered, that is, how the underlying IoT platforms push their resources’ descriptions and the generated observations. RM entry point is tightly coupled with the underlying IoT Registry semantic storage database structure, as depending on the origin and nature of the data, the storage reference differs. Initially the RM has to only choose between the resources or observations graphs.

RM registration endpoint is defined to be complaint with SPARQL 1.1 Graph Store HTTP Protocol [44]. RDF documents structure is open and may include a great variety of information (e.g., multiple resources’ and observations’ annotated descriptions, additional metadata like frequency, availability, quality-related features, etc.). Thus, the first duty for the RM is to guarantee that documents suit the minimum requirements. Therefore, RM must analyze and validate the annotated descriptions. Firstly, the RM checks the compliance with the ontology that is used. It inspects the semantic content to determine the type of data that is being registered and it verifies whether it includes the necessary concepts according to the cardinality expressed in the ontology. In order to avoid delays, due to internal queries, and to ease the process, the registration endpoint is defined as a REST API that replicates the basic IoT Registry database structure. Thus, underlying platforms have to use the proper endpoint when pushing new resources or observations.

Secondly, the RM substitutes the URIs of the triplets that arrive from the underlying platforms replacing them by other URIs that are under a common namespace. These new URIs are also valid URLs as they will be using the IoT Registry namespace. This way, the two conditions for the Cyberphysical Systems as a Service design consideration are fulfilled. On the one hand, the RM detaches the information from its originating platform, thus achieving the platform agnostic paradigm. On the other hand, the WoT-paradigm is also enabled, as every concept in the stored graphs will have a dereferenceable URL.

RM does not only forward the RDF documents submitted by the underlying platforms towards the IoT Registry’s TDB but also exposes read endpoints for each of the semantic subjects within these documents. On each URL, the semantic model of that node can be read. This model will contain the nodes to which that particular node is connected and the relations to each of them. Following the links referenced in the URIs of those neighbor nodes, it would be possible to progressively browse through the entire semantic graph. This behavior extends the HATEOAS (Hypermedia as the Engine of Application State) constraint of a REST API, but in this case applied to semantically described data.

Besides, IoT Registry, through the RM, also provides generic reading endpoints for retrieving the list of IoT resources and observations IRIs. Furthermore, using query parameters it is possible to filter out specific information (i.e., by phenomenon or unit of measurement). This RM endpoint aims at enabling access to semantic information in a more familiar way for traditional web developers, hiding the complexity of dealing with SPARQL queries.

Summarizing, the RM makes it possible to use standard HTTP and REST API procedures to access the semantically annotated documents stored in IoT Registry, wrapping the internals and complex semantic requests used between the RM and the TDB.

### 4.3. Semantic Data Query Endpoint

Taking into account that the IoT registry stores semantically annotated documents into a semantic graph, it is essential that it provides an interface to make semantic queries. SPARQL is known to be the most common and widely used RDF query language. For this reason, we chose to export the semantic query functionality by enabling a direct SPARQL endpoint conformant with SPARQL protocol for RDF [45].

The default endpoint operates on the Global graph, which merges both resources and their gathered observations. Nevertheless, SPARQL queries can be limited to only one of the underlying graphs by using other specific endpoints provided. The underlying methodology is based on the use of FROM clause that allows to reference specific RDF datasets, instructing the SPARQL processor to take the union of the triples from all referenced graphs as the scope of the SPARQL sentence execution. As internal graphs structure and naming are not made publicly available, external users must use the defined interfaces in order to successfully access the whole datasets. 

IoT Registry restricts the execution of SPARQL queries willing to modify the stored content (INSERT, UPDATE, or DELETE). As this endpoint is foreseen to be used by context information consumers, this behavior does not limit the normal operation.

The IoT registry implements an additional functionality related to semantic data query. It provides a repository of SPARQL queries. The queries stored can be static or they can be templates that allow dynamic behavior of the query by assigning values to predefined variables. This repository is designed with the purpose of sharing knowledge between users and smoothing the learning curve of using the platform as a whole. In order to keep the system secure, protection to injection attacks has been implemented.

As it has been described, the semantic data query endpoint extends the functionalities of the RM, providing a more flexible interface towards IoT Registry’s TDB.

### 4.4. Resource Broker

The RDF datasets stored in IoT registry are mainly static semantic descriptions. SPARQL update request are required in order to modify the triples. However, these documents can also include metadata or references to external endpoints that complement the basic information or provide a more updated version.

As it has been already introduced, an important aspect of the IoT modeling that we have followed is that IoT devices can directly export their services. Every device can define multiple instances of the class *iot-lite:Service* (iot-lite namespace is defined as http://purl.oclc.org/NET/UNIS/fiware/iot-lite#), whose properties include a service endpoint (URL). For instance, this service can refer to the last observation of the sensor, which will be directly accessible through the URL provided, or, for the case of an actuator, it can refer to how to enable one of its functionalities. The Resource Broker (RB) is the component in charge of enabling the access to IoT devices’ services while keeping the required platform agnostic nature and homogenizing the way of accessing them for the end user also.

The RB is also relevant from a security point of view. Underlying platforms will delegate access control to their sensors and actuators to the IoT Registry who, based on its own registered users and groups, and the profiles and policies defined for them by agreement with the platform owner, will in turn enable the path to the end device. The namespace transformation implemented by the RM also applies to the services exported by IoT devices. Thus, RB will be acting like a proxy that intercepts any request made to any IoT Registry endpoint URL, translates it to its original value and finally forwards it to the corresponding platform endpoint. The RB will also transform the URIs and other relevant values included in the reply. The process is carried out internally, so that it is completely transparent for the end user.

## 5. Semantic IoT Registry Implementation Details

As it has been previously introduced, the IoT Registry is the cornerstone component within a platform that is currently supporting the federation of eleven IoT different platforms [15]. In this sense, besides the design considerations, it is critically important to describe in detail the implementation path followed for some of the aforementioned design features. Moreover, the implementation details depicted in this section are the result of challenging the developed system against scalability and heterogeneity issues that only a federation with over 10,000 IoT devices that generates some hundreds of thousands observations daily can demand.

### 5.1. URI Flatten Process

The Semantic Web is all about making links between datasets understandable not only by humans but also by machines. When semantically describing entities, a unique identifier in the form of an URI is usually assigned. However, it is not mandatory that this identifier is dereferenceable on the web. 

This situation applies to the data supplied to the IoT Registry by the federated platforms. To address this issue, the RM transforms original platforms’ URIs into URLs associated to a common and dereferenceable namespace that can be referenced by any web application. We called this process URI flattening. The procedure pursues a twofold objective: on the one hand, enabling web references to semantic entities; and on the other hand, fulfilling the platform agnostic paradigm. The latter is achieved by hiding the binding with the source platform when renaming the entity.

For the RB to properly proxy the queries to the services exported by the underlying IoT devices, it was necessary for the process to be reversible. This is, to be able to go from the flattened URI to the original one and vice versa. In order to avoid the potential delays when accessing a big look-up table storing all the mappings, we implemented an algorithm based on symmetric cryptography. The flattened URI results from ciphering the original URI for generating its corresponding flattened URL. This solution allows us to quickly go from and back to the original URI by just knowing the secret key.

**Figure 3:**
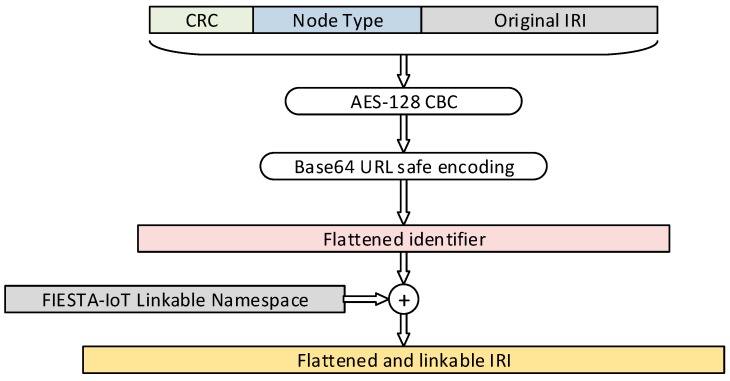
Uniform resource identifier (URI) transformation algorithm.

Figure 3 shows the process implemented within the RM for the transformation of the URIs for all the nodes stored at the TDB graph database.

1. The original URI is prepended by a Cyclic Redundancy Check (CRC) or a short summary of any hash function made over the original URI and an integer which represents the entity; and the entity type, which is an identifier of the entity (e.g., testbed entities are represented with the NodeType 0x03).
2. The resulting string is cyphered using AES-128 block-cypher and Base64 URL safe encoded.
3. The resulting string is appended to the corresponding common IoT namespace resulting in the corresponding transformed URI.

The values prepended to the original URI are used not only for integrity check, but also to randomize the beginning of the resource URL, so it is harder to determine the source testbed. Otherwise, as we are using a block cypher mechanism, entities with the same namespace will have similar URLs. In order to increase the randomness of the resulting URL, it is possible to include a salt also.

Table 1 includes one example of the transformation process. The procedure is applied before storing triples in the TDB to instances of classes, either subjects or objects of the RDF statements, but also to some specific literals, based on the related property. In this sense, we mainly consider the modification of literals whose value is a URL or a direct reference to an exported service. For example, instances of *iot-lite:Service* class can define a property *iot-lite:endpoint*, which is defined as *xsd:anyURI*, and usually it is a URL where the corresponding service is available. This is closely related to the RB functionality introduced above.

**Figure 4:**
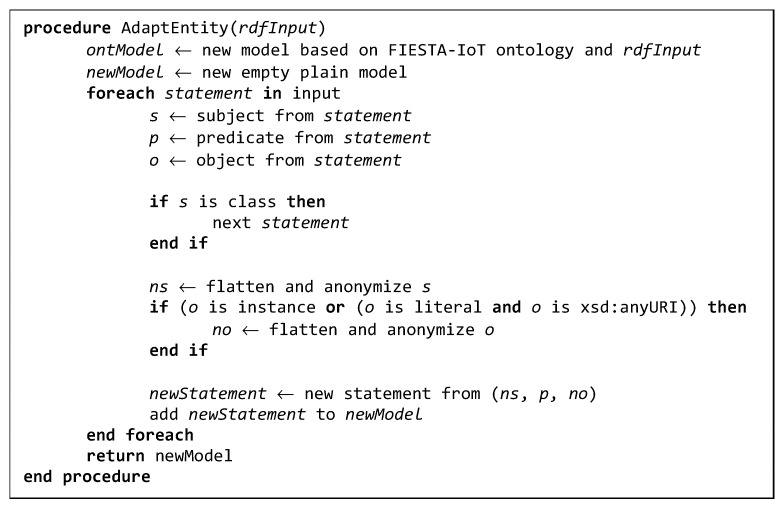
Transformation procedure pseudocode.

Therefore, the RM has to analyze all the triples posted by testbeds in order to identify classes instances and, for FIESTA-IoT specific case, *iot-lite:endpoint* references. Implementation-wise, we can rely on several SPARQL queries and the subsequent generation of the new semantic model. However, we have opted to take advantage of Jena functionalities, especially those related with OntModel, as it provides a better integration and coding experience. Figure 4 shows the pseudocode of the procedure.

The main premise of the procedure is that every subject has to be dereferenceable unless it is a class definition. It does not matter whether it is blank or named node. Then, when we iterate over each RDF statement of the semantic document, we transform every subject following the previous premise. Besides, for every object we check whether the value is a reference to a class, to a class instance or to a URL literal and perform the same operation.

In order to be able to achieve this, we initially generate an OntModel from the original posted RDF document. We use this new model to check or infer the nature of each RDF statement subject and object. We also filter some properties and cache IRIs in order to reduce the inference request and speed up the process. The properties that are filtered are those 

### 5.2. Semantic Document Content Validation

A particularly interesting aspect of using semantics is the ability to validate not only syntactically but also semantically the data [46]. However, even if the IoT registry could be guaranteed that documents posted by the underlying IoT infrastructures to respect the ontology employed, this is not enough to completely prevent from the injection of graph inconsistencies that might lead to the storage of loose data. RDF documents are quite flexible in terms of the data they include. For instance, registering a resource description that does not include the proper bond with its associated platform, or an observation not including a reference to the node that was generated it or its value and unit of measurement, will make the information provided not fully useful. Syntactically and semantically, these two examples can perfectly pass the filter but they would still lead to nodes in the graph that are not properly bound to the WoT. Even if the generator is willing to provide the full description, but as two separate and independent RDF documents, IoT registry cannot take the risk of not receiving one of the pieces.

In order to avoid this, the IoT registry carries out an additional validation step, in this case, for making sure that the resource(s) or observation(s) do carry all the information required for describing a resource or recording a measurement from a sensor appropriately. To do this, the module runs internally several queries that will check not only that it contains all the mandatory nodes for a resource description or an observation graph, but also that the required properties are present (as stated by the properties’ cardinality at the ontology definition).

As a single RDF document can contain more than one resource or observation, it is important that the process implemented checks for every resource or observation reference in the RDF description.

As it has been previously mentioned, we have used the FIESTA-IoT ontology [43] as the basis for the reference implementation that we have developed. In this sense, we consider that a resource description must include a bond to the IoT platform to which it belongs, its location, and the measured phenomenon along with its unit of measurement. Similarly, an observation description must consist of, at least, its value, unit and quantity kind, the location, and timestamp when it was generated and a link to the sensor. Additional information might be interesting to have, but this enumeration is considered the bare minimum to accept a RDF description.

**Figure 5:**
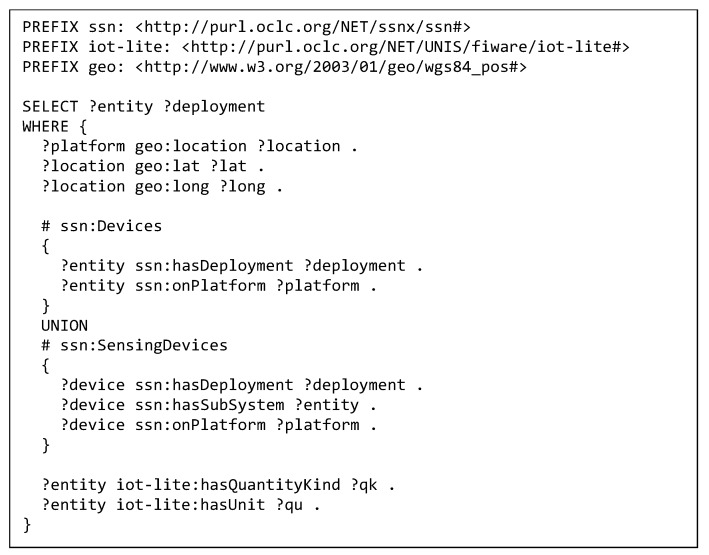
Example of minimal document validation for a resource.

Figure 5 shows an example of a minimal document validation SPARQL. This SPARQL query is applied to the RDF document posted, and no interaction with IoT registry’s TDB is required. The outcome of the execution of the SPARQL sentence is a list of resources whose description meets the minimum set of information as defined by the FIESTA-IoT ontology. Besides, another SPARQL query extracts the list of linked resources within the RDF document; both lists must match. Otherwise, it would mean that the RDF document is including invalid descriptions. Once this first check is passed, it is verified that the deployments or IoT platforms associated are already registered in the TDB. Initially, and based on the semantic approach taken, it could seem that the simplest way this can be done is through the execution of another SPARQL request on the TDB. However, in order to minimize the amount of time spent on the validation procedure and reduce the amount of tasks run on the TDB, we have taken an alternative path. As the list of underlying platforms registered can be considered quite static and not very large, we can keep it either in memory or in a relational database. Then, matching the presence of platforms in both lists is straightforward.

This way, the content validation process for resource registration is done as a fully separate process, without interfering in the read and write operations on the TDB or the IoT registry’s SPARQL execution engine. The same approach is applied for observations implementing the required adaptations in the process. Particularly, modifying the SPARQL query and testing that the associated resources are already registered.

Upon execution of the content validation procedure, and considering that the answer is not empty, we can assert that the information is valid and we can proceed to store it into the TDB. Otherwise, the full document is rejected, informing the user about the resources or observations that are not properly described.

It is worth mentioning that this process introduces a non-negligible computational overhead, as every request implies the execution of an internal SPARQL query. However, we consider that the benefit outweighs the expected registration delay as we have control over the data really stored and its usefulness. What is more, as described, it runs as a fully independent feature, not jeopardizing TDB performance.

### 5.3. TDB Organization Evaluation

IoT registry’s triplestore stores semantic information that is grouped in two realms, resources and their observations, and as such, the TDB is organized. The dataset was divided in two named graphs keeping the IoT devices and the observations that they generate in two independent graphs that were only logically linked through the IoT device identifier. 

It is clear that the difference in size between these two graphs can become large. The Resources graph is almost static since underlying platforms only have to register their assets once, at the very beginning, with small one-time updates when incorporating new resources. However, as long as sensors keep pushing data, the Observations graph will never stop growing, becoming difficult to handle.

Using one large graph for all the observations would make that even the simplest SPARQL queries had scalability problems. Thus, in order to mitigate the effect of Observations graph growth and to improve system performance, we came up with a solution that consists on splitting the graph into a number of subgraphs. Even though technically speaking, the IoT registry keeps saving all the observations into the TDB, the usage of various and independent named graphs can hold the information isolated when it comes to process SPARQL queries. This way, the system will have to seek into a portion of the dataset, limited to only selected graphs. 

**Figure 6:**
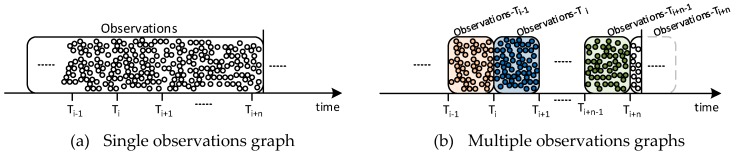
Single- vs. multi-observation graphs.

Figure 6 depicts the proposed solution. We graphically represent, first in Figure 6a, how observations (represented as bubbles) are stored into either a standalone graph or, on the other hand, in Figure 6b, sliced into various subgraphs, whose name can include indexing information that helps on accessing the stored information.

In our case, we consider time as the basis for the generation of new subgraphs. Another option would have been grouping the observations by resource, but this would only have helped when fetching information from one specific sensor. However, typically information is requested per location and period. The subgraph’s creation time is appended to graph name to create a unique subgraph identifier. These subgraphs are created periodically and the interval between two consecutive graphs is fixed and preconfigured. For instance, a name like *observations:201803151316* corresponds to the subgraph created at 2018/03/15 13:16 UTC. This graph will store all observations posted to IoT Registry from that time on during the fixed interval.

From the end user standpoint, the existence of multiple subgraphs is mostly hidden. The REST API that gives access to RM and Semantic data query engine includes two query parameters (*from* and *to*) to set the time constraints of the underlying query, that is, to define the FROM statements to be included to the SPARQL queries. By fixing these parameters, the consumer of context information stored at the IoT Registry can directly specify the time interval they want to focus on, instead of having to perform an exhaustive search onto the whole TDB. If none of these parameters is present, then the query is only solved against the observations stored in the latest subgraph. 

**Figure 7:**
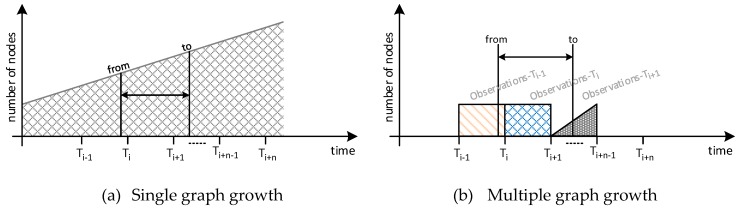
Single- vs. multi-observation graph(s) operation.

Figure 7 presents the reduction in the total number of RDF statements per graph of the proposed solution. Following the previous example, where a SPARQL query to retrieve observations in a specific time interval would search into the whole graph (see Figure 7a), with the implemented configuration, the IoT registry only attach graphs *observations-T_i−1_*, *observations-T_i_*, and *observations-T_i+1_* to the process (Figure 7b), significantly reducing the time and complexity of the search.

It is important to note that it is not possible to guarantee that all observations stored in a time-based subgraph have been taken in the corresponding period, as the observations are stored as per time of arrival not as per generation time. This latter approach would imply that the timestamp included in its semantic description would be checked and it would introduce some non-negligible delay in the observation processing time. Hence, the correspondence between the observation’s time and subgraphs’ name is highly dependent on underlying platforms good practices. Data consumers have to take into account this potential lack of synchronization between the interval included in the request and actual timestamps of the measurements.

Summarizing, thanks to the configuration described, the system experiences various improvements that, altogether, lead to a better overall performance:

1. Since most of the queries address recent data, (i.e., typically only last values are of interest due to the stream nature of IoT) the reduction in size of the target graph enables a much more agile response. Thus, with respect to an end user, the quality of service, and experience is significantly enhanced.
2. Extrapolating to a general point of view, operations on reduced datasets mean less computational load. As a number of users will be interacting with the platform at the same time, the shorter the time dedicated per request, the higher the number of requests that can be processed without saturating the system.
3. Access to historical data is still possible for those data consumers that require information beyond the most recent context data. For those users, the capacity to sequentially query against consecutive time intervals allows them avoiding queries that would result excessively heavy if made over the compounded period.

## 6. Conclusions and Open Issues

Internet of Things testbed deployments have been growing at an incredible pace over the last few years, although ontologies and access tools are not yet fully standardized for IoT resources. Therefore, each testbed provides its own solution to access them. In this paper, we have presented the IoT Registry—an enabler of the Semantic Web of Things—which address data access problematic across heterogeneous IoT testbeds, providing a common semantic-aware homogenous access to their resources.

The IoT registry is a fully-fledged warehouse that stores all the data injected by different testbeds that belong to the FIESTA-IoT federation [15]. On top of this repository-like behavior, it provides several means of access for experimenters to collect testbed’s data, where all resource descriptions and observations are provided in a testbed agnostic approach. Upon the scalability issues brought about by the huge amount of data pushed by these IoT testbeds (tens of thousands of observations per day), we have introduced a solution that with a simple-yet-effective time-slicing approach, the overall system performance is maintained below acceptable margins [42].

Among the future steps envisioned, we plan to carry out a thorough assessment on all IoT Registry’s operations in order to characterize its behavior. Furthermore, we would also like to compare its performance with that of other mainstream platforms, such as FIWARE and OneM2M, which are called to stand out as the future references for IoT and M2M. Additionally, some policy has to be settled for long-term historical datasets and at some point, this data should not be available through the IoT Registry but on another kind of platforms (more oriented to Big Data than to WoT). Even when the IoT Registry implementation described in this paper has been running from beginning of 2017 till the end of 2018 and, during this period, it has been able to support several experiments (FIESTA-IoT Project Experiments (http://fiesta-iot.eu/index.php/fiesta-experiments/)) on different application domains, from scalability and usability point of view, it is not appropriate to store data at the IoT Registry forever. During the IoT Registry aforementioned operation time (almost two years), disk space was not a particularly challenging aspect, since storage space is quite cheap at the cloud, and, precisely, the solutions that we have implemented have allowed reasonably good behavior even under complex real scenario.

## Figures and Tables

**Table 1:** URI flattening process example.

Testbed	FIESTA-IoT URL
Original IRI	http://api.smartsantander.eu#SmartSantanderTestbed
Entity type	0x03 (Testbed)
CRC	0x50A32758
IoT Registry URL	https://platform.fiesta-iot.eu/iotregistry/api/testbeds/kscYbDJBhbywRuRSSOsucfEhrY1lTb5LF6bYBh36pTbvKDqUIfDkS7WeB9ryaC7l-C9ZExZYLwiyuw8wAKjZpQ==
